# Ultrasensitive barocaloric material for room-temperature solid-state refrigeration

**DOI:** 10.1038/s41467-022-29997-9

**Published:** 2022-04-28

**Authors:** Qingyong Ren, Ji Qi, Dehong Yu, Zhe Zhang, Ruiqi Song, Wenli Song, Bao Yuan, Tianhao Wang, Weijun Ren, Zhidong Zhang, Xin Tong, Bing Li

**Affiliations:** 1 Institute of High Energy Physics, Chinese Academy of Sciences, Beijing, 100049 China; 2 Spallation Neutron Source Science Center, Dongguan, 523803 China; 3 Shenyang National Laboratory for Materials Science, Institute of Metal Research, Chinese Academy of Sciences, 72 Wenhua Road, Shenyang, 110016 China; 4 School of Materials Science and Engineering, University of Science and Technology of China, 72 Wenhua Road, Shenyang, 110016 China; 5 Australian Nuclear Science and Technology Organisation, Lucas Heights, New South Wales 2234 Australia

**Keywords:** Materials for energy and catalysis, Energy science and technology, Energy

## Abstract

One of the greatest obstacles to the real application of solid-state refrigeration is the huge driving fields. Here, we report a giant barocaloric effect in inorganic NH_4_I with reversible entropy changes of $\Delta {S}_{{P}_{0}\to P}^{{{\max }}}$ ∼71 J K^−1^ kg^−1^ around room temperature, associated with a structural phase transition. The phase transition temperature, *T*_t_, varies dramatically with pressure at a rate of d*T*_t_/d*P* ∼0.79 K MPa^−1^, which leads to a very small saturation driving pressure of Δ*P* ∼40 MPa, an extremely large barocaloric strength of $\left|\Delta {S}_{{P}_{0}\to P}^{{{\max }}}/\Delta P\right|$ ∼1.78 J K^−1^ kg^−1^ MPa^−1^, as well as a broad temperature span of ∼41 K under 80 MPa. Comprehensive characterizations of the crystal structures and atomic dynamics by neutron scattering reveal that a strong reorientation-vibration coupling is responsible for the large pressure sensitivity of *T*_t_. This work is expected to advance the practical application of barocaloric refrigeration.

## Abstract (web summary)

A small driving pressure is desirable for practical application of barocaloric materials. Here, the authors demonstrate a sensitive barocaloric effect in NH_4_I due to strong reorientation-vibration coupling.

## Introduction

To tackle climate change and realize the United Nations’ Sustainable Development Goals, the first priority should be given to the decarbonization of heating and cooling sectors^1^. Nowadays, vapor-compression technology is extensively employed for civil and industry refrigeration, which leads to two serious environmental concerns. On the one hand, billions of running fridges, air conditioning, and heat pump units are swallowing ∼25–30% of the electricity, and this demand is expected to continuously grow by several times in the upcoming decades^2,3^. On the other hand, currently used refrigerants have a thousand-time stronger global warming potential compared to CO_2_^4^. For example, the global warming potential of the popular R134a is about 1300 times higher than that of CO_2_. Given that such refrigerants with good performance but low global warming potential are very limited^4^, it is urgent to establish a low-carbon refrigeration solution.

Within such a context, solid-state refrigeration technology based on caloric effects becomes a promising alternative. Caloric effects usually include magnetocaloric^5^, electrocaloric^6^, elastocaloric^7^, and barocaloric effects^8^, which characterize the thermal effects during a solid-state phase transition induced by a specific external field, such as magnetic field, electric field, stress, and pressure, respectively. In the entire refrigeration process, the working material stays solid and thus this technology is emission-free and compact^9–11^. As far as the energy efficiency is concerned, cooling systems working with caloric materials are considerably competitive as expected to reach 60–70% of the Carnot limit or even be 150% more efficient than the vapor-compression refrigeration from the aspect of thermodynamic coefficient of performance^12,13^.

However, one of the greatest obstacles to the large-scale application of caloric cooling technology is the difficulty that large caloric effects can be only achieved under huge driving fields in current leading materials. For instance, the magnetic fields used to stimulate metamagnetic or magneto-structural transitions in magnetocaloric materials are generally larger than 2 T, which requires heavy and expensive rare-earth-based permanent magnets or superconducting magnets^14^. With respect to the electrocaloric materials, the electric fields are in the magnitude of kV m^−1^ or even MV m^−1^, which might create breakdown phenomena and hence influence the operation reliability and cycling lifetime^15^. In the case of leading elastocaloric materials, the typical driving stress is as large as 700 MPa to obtain good refrigeration performances^16^. As for barocaloric materials, the required pressure is usually above 200 MPa for most intermetallics^17,18^ and it is reduced down to about 100 MPa in the recently discovered plastic crystals^8^. Nonetheless, the development of excellent caloric materials with a smaller driving field remains highly challenging.

In this paper, we report a giant barocaloric effect around room temperature in a commercially available ammonium iodide (NH_4_I) compound. The phase transition temperature in NH_4_I displays high sensitivity to driving pressure, which renders a very small saturation driving pressure and makes NH_4_I one of the most efficient and cost-effective caloric materials as estimated by barocaloric strength (maximum isothermal entropy change normalized by driving force). In addition, thorough studies on crystal structures and atomic dynamics using neutron scattering techniques demonstrate that the excellent barocaloric effect is mainly attributed to the configuration entropy changes of [NH_4_]^+^ tetrahedra in the frameworks formed by I^−^ ions as well as the large sensitivity of phase transition temperature to external pressure due to the strong coupling between molecular reorientations and lattice vibrations.

## Results

### Barocaloric effect in NH_4_I

**Fig. 1:**
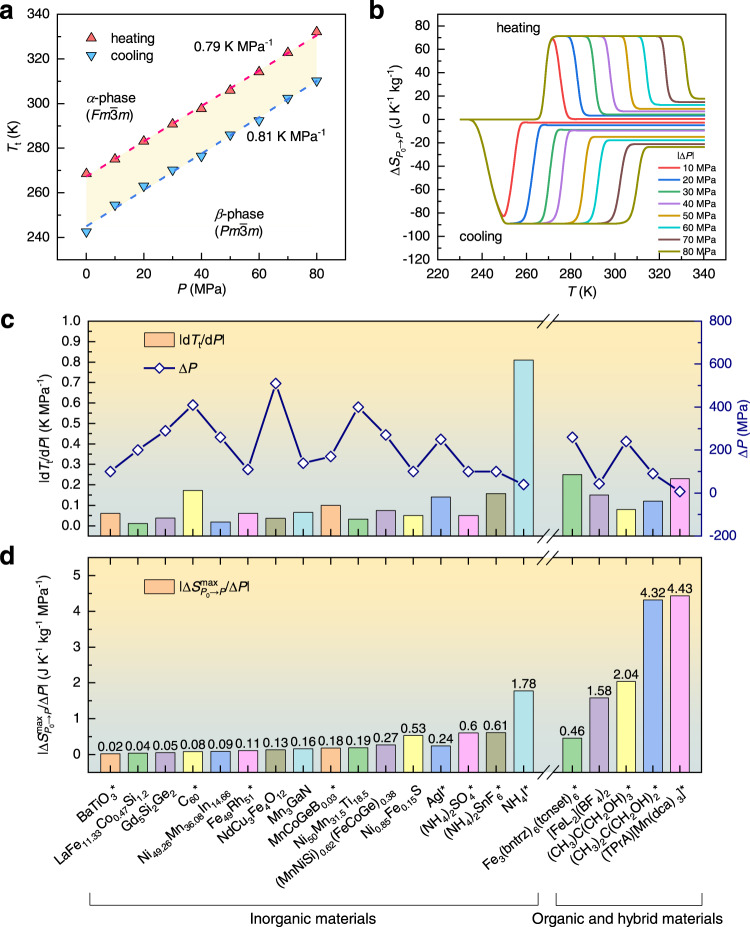
Barocaloric effects of NH_4_I. **a** Phase diagram of NH_4_I as functions of temperature and pressure. The cooling (down-triangle) and heating transition (up-triangle) temperatures are derived from calorimetric data in Supplementary Fig. 2a. **b** Pressure-induced isothermal entropy change, $\Delta {S}_{{P}_{0}\to P}$, for the cooling and heating processes. **c** Much larger pressure-dependent transition temperature variation $\left|{{{{{\rm{d}}}}}}{T}_{{{{{{\rm{t}}}}}}}/{{{{{\rm{d}}}}}}P\right|$ and smaller saturation driving pressure compared with other giant barocaloric materials^8,17,18,23–39^. **d** High barocaloric strength as estimated through $\left|\Delta {S}_{{P}_{0}\to P}^{{{\max }}}/\triangle P\right|$^34^. The data for the samples marked by ‘*’ represents the reversible values with the influence of thermal hysteresis excluded. Details are summarized in Supplementary Table 1.

The barocaloric effect in NH_4_I is studied utilizing differential scanning calorimetry measurements over the temperature range of 230–340 K, under several constant external pressures (Methods and Supplementary Fig. 2). Following the heat flow data (Supplementary Fig. 2a), a phase diagram is established. As shown in Fig. 1a, a phase transition is observed at ∼243 K on cooling or ∼268 K on heating under ambient pressure, close to ∼257 K obtained by heat capacity measurement^19^. A large thermal hysteresis of $\Delta {T}_{{{{{{\rm{hys}}}}}}}$ ∼25 K indicates the first-order nature of this phase transition. This phase transition takes place between the intermediate-*T β*-phase (space group ${Pm}\bar{3}m$) and the high-*T α*-phase (${Fm}\bar{3}m$)^20^. It is found that the phase transition temperature varies strongly with external pressure. Quantitatively, the steep phase boundary is defined by ${{{{{\rm{d}}}}}}{T}_{{{{{{\rm{t}}}}}}}/{{{{{\rm{d}}}}}}P$ ∼0.81 K MPa^−1^ on cooling while ∼0.79 K MPa^−1^ on heating. Although this value is larger than ∼0.43 K MPa^−1^ obtained with nuclear magnetic resonance measurement^21,22^, it is consistent with other thermodynamic and lattice data in this work (next section). It is worth noting that the ${{{{{\rm{d}}}}}}{T}_{{{{{{\rm{t}}}}}}}/{{{{{\rm{d}}}}}}P$ are much larger than those of other leading barocaloric materials as summarized in Fig. 1c^8,17,18,23–39^.

Based on the heat flow data, we obtained the isobaric entropy changes ∆*S*_t_ at the phase transition shown in Supplementary Fig. 2b as well as the pressure-induced entropy changes $\Delta {S}_{{P}_{0}\to P}$ shown in Fig. 1b. Here, *P*_0_ is the ambient pressure while *P* is the applied pressure, as described in the previous report^8^. The maximum (or saturated) value of $\Delta {S}_{{P}_{0}\to P}\left(T,P\right)$ on heating is ∼71 J K^−1^ kg^−1^, which could be realized by a small driving pressure of 40 MPa as shown in Supplementary Fig. 3. This value of $\left|\Delta {S}_{{P}_{0}\to P}^{{{\max }}}\right|$ is comparable to those of other state-of-the-art barocaloric materials (Supplementary Fig. 4a)^8,17,18,23–39^. The entropy changes in the unit of J K^−1^ cm^−3^ are also plotted in Supplementary Fig. 4b. The value of 0.21 J K^−1^ cm^−3^ for NH_4_I is also among the largest in inorganic materials. Moreover, the giant ${{{{{\rm{d}}}}}}{T}_{{{{{{\rm{t}}}}}}}/{{{{{\rm{d}}}}}}P$ value also opens a wide reversible working temperature window of ∼41 K under 80 MPa (Supplementary Fig. 3). The pressure sensitivity also gives rise to a giant barocaloric strength, defined by the maximum entropy changes normalized by the saturation pressure, $\left|\Delta {S}_{{P}_{0}\to P}^{{{\max }}}/\triangle P\right|$, which is ∼1.78 J K^−1^ kg^−1^ MPa^−1^. As summarized in Fig. 1d, the barocaloric strength of NH_4_I is much larger than most other barocaloric materials, especially compared with the inorganics. Based on the entropy changes and specific heat capacity^40^, we also estimate the adiabatic temperature via the formula $\Delta {T}_{{{{{{\rm{ad}}}}}}}=\left|T\Delta {S}_{{P}_{0}\to P}/{C}_{P}\right|$^41^, which is ∼34 K. This is almost ranked as the biggest among the state-of-the-art barocaloric materials as summarized in Supplementary Fig. 4c and Table 1.

In addition to the isobaric measurements, the direct measurements of pressure-induced heat flow were also carried out at 298 K, as shown in Supplementary Fig. 5. Obvious exothermic and endothermic peaks are observed within pressurization (50 → 90 MPa) and depressurization (50 → 7.5 MPa) processes, respectively. The pressure-induced entropy changes are estimated as 62.7 and 65.6 J K^−1^ kg^−1^ for the pressurization and depressurization processes, respectively. These values are in good agreement with the value of 71 J K^−1^ kg^−1^ from heat flow measurements under constant pressures shown in Fig. 1.

### Phase transitions as a function of temperature

As the origin of the observed barocaloric effect, the phase transition is considered in aspects of crystal structures, reorientation dynamics, and lattice dynamics. According to previous reports, NH_4_I undergoes successive phase transitions from low-*T* tetragonal *γ*-phase ($P4/{nmm}$) to intermediate-*T* cubic *β*-phase (${Pm}\bar{3}m$), and then to another cubic *α*-phase (${Fm}\bar{3}m$) (schematic crystal structures are shown in Supplementary Fig. 6)^20,42^. Our temperature-variable X-ray diffraction (XRD) measurements confirm a first-order phase transition on heating (Supplementary Fig. 7a). The diffraction patterns can be indexed with the *β*-phase below *T*_t_ and *α*-phase above *T*_t_, respectively. The temperature dependences of lattice parameters are shown in Supplementary Fig. 7b, c. The slope of the temperature dependence of unit cell volume, ${\left(\partial V/\partial T\right)}_{{P}_{0}}$, is determined to be 3.3 × 10^−8^ and 4.23 × 10^−8^ m^3^ kg^−1^ K^−1^ for *β* and *α* phases, respectively. As a result, the lattice contractions under pressure have a marginal contribution to the total entropy changes for both phases, less than 0.4 J kg^−1^ K^−1^. In addition, the volume change across the *β*↔*α* phase transition under ambient pressure is determined to be ∆*V*_t_ ∼5.87 × 10^−5^ m^3^ kg^−1^ or 16.95%, which is in good agreement with the value of 16.96% in the literature^43^. Following the Clausius-Clapeyron equation $\triangle {S}_{{{{{{\rm{t}}}}}}}=\triangle {V}_{{{{{{\rm{t}}}}}}}/\left(\frac{d{T}_{{{{{{\rm{t}}}}}}}}{{dP}}\right)$, this change corresponds to an entropy change of 74.3 J kg^−1^ K^−1^, which also agrees well with the experimentally determined value of ∼71 J kg^−1^ K^−1^ in Supplementary Fig. 2.

**Fig. 2:**
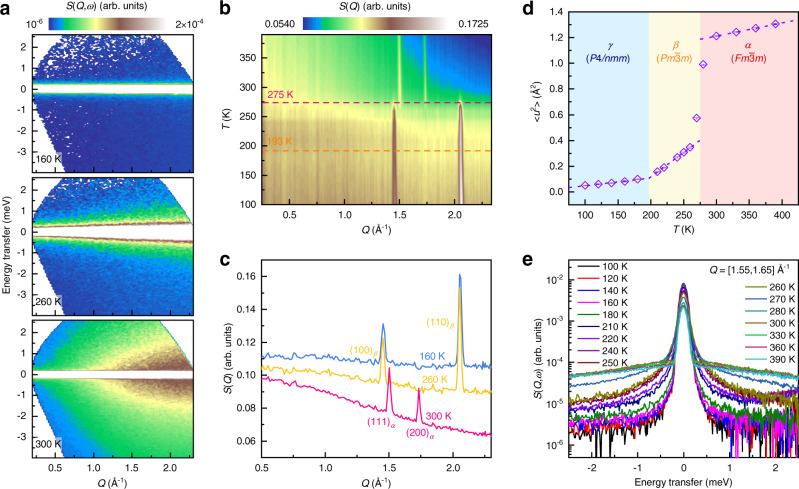
Phase transitions as a function of temperature in NH_4_I. **a** The contour plots of dynamic structure factor, *S*(*Q*,*ω*), for NH_4_I at 160, 260, and 300 K measured at PELICAN with *E*_i_ = 3.72 meV. **b** The contour plot of elastic structure factor *S*(*Q*) as a function of temperature. **c** Comparison of *S*(*Q*) at 160, 260, and 300 K. The Bragg peaks for the high-*T α*-phase and the intermediate-*T β*-phase are marked with subscripted ‘*α*’ and ‘*β*’, respectively. **d** Experimental mean-squared displacement, <*u*^2^>, determined from the fitting of *S*(*Q*) to *S*(*Q*) ∝ exp(−*Q*^2^〈*u*^2^〉/3) (see Supplementary Fig. 8). **e** Sliced *S*(*Q*,*ω*) over the *Q* range of [1.55, 1.65] Å^−1^ as a function of energy transfer.

For atomic dynamics, the dynamic structure factor $S(Q,\omega )$ is obtained using inelastic neutron scattering (INS) measurements as a function of energy transfer (*ω*) and momentum transfer (*Q*) with the Time-of-Flight Spectrometer, PELICAN, at the Australian Centre for Neutron Scattering (see Methods)^44^. Figure 2a illustrates three typical INS spectra, $S(Q,\omega )$, collected at 160, 260, and 300 K, which correspond to the three phases, respectively. These spectra exhibit different features. At first, the elastic component *S*(*Q*) is extracted by integrating $S(Q,\omega )$ over [−0.3, 0.3] meV. The results are shown in Fig. 2b, c. An obvious phase transition can be found at ∼275 K, corresponding to the transition from the intermediate-*T β*-phase to the high-*T α*-phase^20,42^. However, it is quite difficult to identify another phase transition between the low-*T γ*-phase and the intermediate-*T β*-phase, as the *γ*-phase is derived from the *β*-phase with a tiny distortion^20,45^.

To accurately track the phase transition, the atomic mean-squared-displacement (MSD) is analyzed with the Debye-Waller factor fitting of the elastic structure factor, *S*(*Q*) (see Supplementary Fig. 8)^46^. The obtained MSD as a function of temperature is shown in Fig. 2d. The MSD across the transition of *β*→*α* exhibits an abrupt jump. In addition, it is noted that the temperature dependences of MSD present a crossover at ∼193 K, which corresponds to the phase transition of *γ*→*β*. Given that the cross-section of H (80.26 barn) is much larger than those of I (0.31 barn) or N (0.5 barn), the obtained MSD in Fig. 2d mainly reflects the thermal fluctuation behaviors of hydrogen atoms. In fact, the sliced *S*(*Q*,*ω*) curves over the *Q* range of [1.55, 1.65] Å^−1^ also show strong broadening above 193 K, and this broadening develops continuously with increasing temperature until 280 K as shown in Fig. 2e. These two temperature points correspond exactly to the phase transition temperatures of *γ*→*β* and *β*→*α*.

### Order-to-disorder transition and reorientation dynamics

**Fig. 3:**
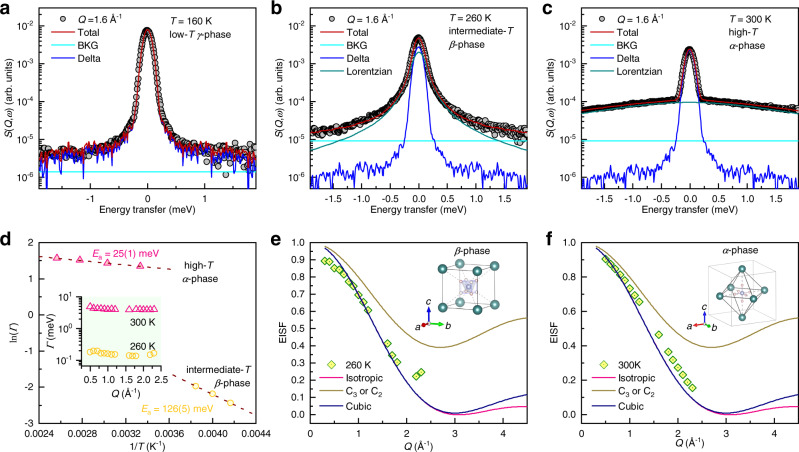
Reorientation dynamics of ammonia molecule. **a**–**c** Spectral fitting of the sliced *S*(*Q*,*ω*) over the *Q* range of [1.55, 1.65] Å^−1^ at (**a**) 160, (**b**) 260, and (**c**) 300 K, respectively. One constant background (BKG) plus a delta function convoluted with the resolution function could make a good fitting for the spectrum at 160 K, while one more Lorentzian profile is needed at 260 and 300 K. **d** Temperature dependence of the full width at half maximum, *Γ*, of the Lorentzian components for the intermediate-*T β*-phase and the high-*T α*-phase, which are fitted to the Arrhenius equation, $\varGamma \propto {{\exp }}\left(-\frac{{E}_{a}}{{k}_{{{{{{\rm{B}}}}}}}T}\right)$, where *E*_*a*_ is the activation energy for the motions and *k*_B_ is the Boltzmann constant. Inset shows *Q* dependences of the *Γ*. **e**, **f** Experimental EISF compared with different reorientation models at 260 and 300 K, respectively. Insets show the schematic diagrams of the coordinations between I^−^ and [NH_4_]^+^ ions in the *β*- and *α*-phases, respectively. Following the symmetric operations, the hydrogen atoms could dwell on any of the diagonal lines in the *β*-phases, while the hydrogen atoms residing along the [100] direction would give a sixfold steric distribution in the *α*-phase (single-approach model, see Supplementary Fig. 6).

The broad peaks centered around 0 meV as observed in Fig. 2a, e are signals of quasi-elastic neutron scattering (QENS). QENS is widely used to study the dynamics of molecular reorientations of hydrogen-contained materials^47^. *S*(*Q*,*ω*) over the *Q* range of [1.55, 1.65] Å^−1^ below 190 K could be fitted with a delta function convoluted with the instrumental resolution function plus a linear background (Fig. 3a). This implies that hydrogen atoms in [NH_4_]^+^ tetrahedra stay in the lattice of the low-*T γ*-phase, without a jump or rotation in the given energy window, which is in agreement with the crystallographic analysis based on diffraction data^20^. In contrast, a good fitting for the spectra above 190 K needs one more Lorentzian component as depicted in Fig. 3b, c, which is indicative of the activated motions of hydrogen atoms in the *β*- and *α*-phases.

The relaxation time, *τ*, of the reorientation modes is estimated by the full width at half maximum (*Γ*) of the Lorentzian profile with the formula of $\tau =2{{\hslash }}/\varGamma$^48^. As shown in the inset of Fig. 3d, the average *Γ* at 300 K is ∼4.09 meV (*τ* ∼0.32 ps), which is ∼25 times smaller than the value of ∼0.16 meV (*τ* ∼8.2 ps) for the 260 K spectrum. Therefore, the reorientation mode in the high-*T α*-phase is ∼25 times faster than that in the intermediate-*T β*-phase, in agreement with the previous reports^49^. It is also noted that *Γ* for both 260 K and 300 K spectra are almost independent of *Q*, suggesting a localized nature of the reorientation modes similar to perovskite CH_3_NH_3_PbI_3_ and nano-NaAlH_4_^48,50^. In addition, the activation energies of the reorientation modes are examined by fitting the temperature-dependent *Γ*(*T*) to the Arrhenius relation. The activation energy of the reorientation modes in the intermediate-*T β*-phase is 126(5) meV, much larger than 25(1) meV for the high-*T α*-phase (Fig. 3d).

The ratio between the elastic intensity (integrated area below the delta function) and the total intensity (elastic intensity plus the QENS intensity below the Lorentzian profile) gives rise to the elastic incoherent structure factor (EISF), whose *Q* dependence reflects the reorientation geometry. Here, several models are employed to reproduce the experimental EISF, including twofold (*C*_2_) and/or three-fold (*C*_3_) jumps, cubic tumbling as well as isotropic rotational diffusion (see Methods for details). As shown in Fig. 3e, f, the *C*_2_ and/or *C*_3_ model can be easily ruled out for EISF at both 260 (*β*-phase) and 300 K (*α*-phase). However, the EISF can be well reproduced with the cubic tumbling and isotropic rotational diffusion models. It cannot make a clear distinction between these two models due to the limited *Q* range of the current data.

The reorientations of [NH_4_]^+^ tetrahedra are restricted by their molecular symmetry as well as the local crystal environment. Therefore, symmetry analysis is also employed for further discussion about the reorientation modes in NH_4_I. In the intermediate-*T β*-phase, the [NH_4_]^+^ tetrahedron resides in a cube cage formed by eight I^−^ ions as illustrated in the inset of Fig. 3e. In this geometry, each N–H bond (three-fold axis) of the [NH_4_]^+^ tetrahedron is aligned with the three-fold axis of the cube cages, so that four N–H···I hydrogen bonds could be built and then lead to an energy minimum. This set leads to a *T*_d_ configuration, and the [NH_4_]^+^ tetrahedra have two orientational freedoms, which match the cubic tumbling model as shown in Fig. 3e^45,51^. In the high-*T α*-phase, the reorientation dynamics become more intricate. Each [NH_4_]^+^ ion is surrounded by six I^−^ ions, which form an octahedral cage. A lot of models have been proposed in early literatures^20,52–54^, such as single-approach, double-approach, and triple-approach models^52^ or isotropic model^54^, although no exclusive decision can be made. One common feature of these models lies in that the four tetrahedrally arranged hydrogen atoms cannot achieve a close approach to the octahedrally distributed I^−^ ions simultaneously^52^. In this work, the single approach is considered, where only one linear N–H···I hydrogen bond is formed (see inset of Fig. 3f or Supplementary Fig. 6c, d). In this configuration, the [NH_4_]^+^ tetrahedron has six orientational freedoms^45,51^. This leads to a configuration entropy change of 63 J K^−1^ kg^−1^ across the *β*→*α* transition (see Methods), close to the entropy change of 71 J K^−1^ kg^−1^ in Fig. 1b.

### Strong reorientation-vibration coupling

**Fig. 4:**
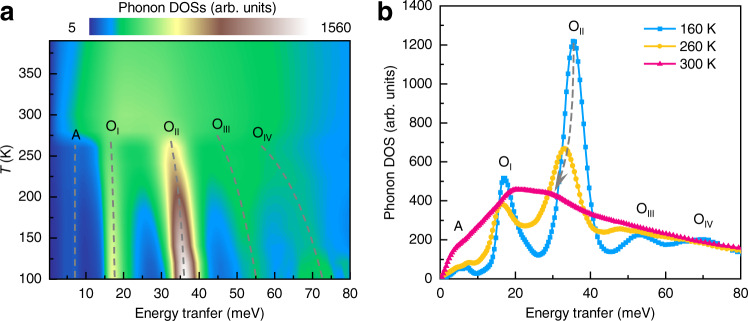
Variation of lattice dynamics with temperature. **a** Contour plot of neutron-weighted phonon density of states (DOSs) as a function of temperature. **b** The profiles at 160, 260, and 300 K, respectively. The dashed lines are used for guiding eyes to track the variation of the phonon bands with temperature. ‘A’ and ‘O’ denote the acoustic and optical phonons, respectively.

The neutron-weighted phonon density of states (DOSs) was measured on the NH_4_I powder sample at PELICAN (Methods), and the results are shown in Fig. 4. The phonon DOS profile at 160 K contains six well-defined peaks up to 80 meV. According to the previous INS studies, the ∼4.8 and ∼7 meV peaks are related to the acoustic (marked as ‘A’) phonon bands, and the ∼18 meV peak is associated with the optical (marked as ‘O’) phonon band^55,56^, while the other three optical phonon bands at higher energy range have not yet been reported. However, the corresponding counterparts have been observed in NH_4_Br^57^, although the peak positions exhibit some differences because of the different molecular masses or chemical bonding strengths in NH_4_I and NH_4_Br. Thus, these three high-energy optical phonon bands would be attributed to the libration motions of the hydrogen atoms in [NH_4_]^+^ tetrahedra.

One obvious phenomenon about the phonon DOSs is the significant broadening at the transition temperature of ∼275 K, above which the profiles become featureless. In addition, it is observed that the optical phonon bands, especially the O_II_, O_III_, and O_IV_ ones, show dramatic softening with increasing temperature as delineated by the dashed lines in Fig. 4. These two features of the phonon DOSs imply that the lattice vibration potentials of NH_4_I are very shallow and anharmonic. In combination with the fact that the hydrogen atoms in [NH_4_]^+^ tetrahedra exhibit different reorientation dynamics with the development of phonon anharmonicity (Fig. 3), it can be concluded that NH_4_I compound has a strong coupling between molecular reorientations and lattice vibrations, perhaps through the hydrogen bonds between the [NH_4_]^+^ tetrahedra and their I^−^ coordination environments. This phase transition dominated by reorientation-vibration coupling in NH_4_I can also be understood from the viewpoint of thermodynamics. With increasing temperature, the rising configurational entropy of [NH_4_]^+^ tetrahedra tends to stabilize the high-*T* phases, similar to the entropy-driven structural transition in formamidinium lead iodide perovskite^58^.

### Responses of dynamic behaviors to external pressure

**Fig. 5:**
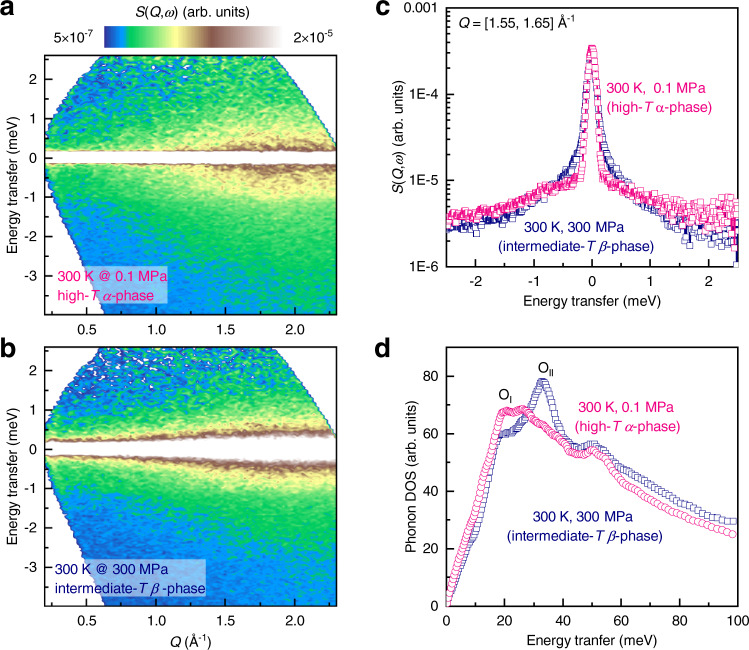
Responses of dynamic behaviors to external pressure. **a**, **b** The dynamic structure factor, *S*(*Q*,*ω*), for NH_4_I at 300 K under external pressure of **a** 0.1 MPa (ambient condition) and **b** 300 MPa, respectively. **c**, **d** Comparison of **c** the *S*(*Q*,*ω*) sliced over *Q* = [1.55, 1.65] Å^−1^ and **d** phonon DOSs between the high-*T* phase (red) and intermediate-*T* phase (blue) in responses to external pressure.

The responses of the phase transition to external pressure are also studied using INS under different pressures. Shown in Fig. 5a, b are contour plots of *S*(*Q*,*ω*) under 0.1 and 300 MPa at room temperature, respectively. This comparison becomes clearer in the sliced curves over the *Q* range of [1.55, 1.65] Å^−1^ as shown in Fig. 5c. The faster (larger *Γ*) reorientation mode is suppressed to be the slower (smaller *Γ*) mode. In addition, with the suppression of the faster reorientation mode, two peaks emerge at ∼19 and ∼34 meV from the featureless phonon DOS of the high-*T α*-phase, corresponding to the O_I_ and O_II_ bands in the intermediate-*T β*-phase as shown in Fig. 5d. It can be seen that the application of external pressure certainly induces changes in both the reorientation and lattice dynamics. These microscopic dynamic responses to pressure provide a solid standing point for understanding the giant barocaloric effect in NH_4_I.

Finally, we would like to emphasize the decisive role of reorientation-vibration coupling in the giant barocaloric effect in NH_4_I. Such a coupling is reflected in the simultaneous variations of crystallographic symmetries, anion coordination, mean-squared displacement, lattice dynamics as well as reorientation dynamics, where the N–H···I hydrogen bonds between [NH_4_]^+^ and I^−^ play the key roles, as suggested in literatures^59,60^. More straightforward, the pronounced optical phonon softening (reduced phonon frequencies) as observed in Fig. 4 is linked to the weakening of the N–H···I hydrogen bonds with increasing temperature. Considering the pressure effect, phonons become hardened due to the compressed lattice volume, which is readily understood even in the quasi-harmonic approximation. At the same time, the reorganized hydrogen bonds with greater strength might suppress molecular reorientations, which gives rise to large entropy changes. The coordination environments are subsequently changed from the six-octahedron configuration to the eight-cube configuration (Supplementary Fig. 6). Consequently, the intermediate-*T β*-phase is induced. The energy scales are manifested as the large increase of the reorientation activation energy from 25(1) meV in the high-*T α*-phase to 126(5) meV in the intermediate-*T β*-phase (see Fig. 3d). Hence, it is plausible that the giant entropy changes mainly originate from the configurational contribution, and the small saturation driving pressure is rooted in the strong reorientation-vibration coupling.

## Discussion

To summarize, we present a thorough study on the inorganic NH_4_I compound which exhibits a giant barocaloric effect over a broad temperature range around room temperature. One of the most noticeable features of NH_4_I is the giant pressure sensitivity of the phase transition temperature. Although larger entropy change requires smaller ${{{{{\rm{d}}}}}}{T}_{{{{{{\rm{t}}}}}}}/{{{{{\rm{d}}}}}}P$ as suggested by the Clausius-Clapeyron equation^33^, a larger ${{{{{\rm{d}}}}}}{T}_{{{{{{\rm{t}}}}}}}/{{{{{\rm{d}}}}}}P$ value is also desirable in practice for smaller driving pressures as demonstrated in NH_4_I. The great pressure sensitivity of the phase transition temperature stems from the strong coupling between molecular reorientations and lattice vibrations, while the giant entropy change is largely contributed by the orientational disorder of [NH_4_]^+^ tetrahedra. This work is expected to inspire the discovery of giant barocaloric materials with high pressure-sensitive phase transition, and hence push a big step forward towards the realization of efficient and affordable barocaloric refrigeration.

## Methods

### Sample preparation and characterization

The NH_4_I powder sample with 99.999% purity was purchased from Aladdin. The XRD measurement was carried out on a powder sample at a Rigaku Miniflex-600 diffractometer with Cu-*K*_α_ radiation over 10°–90° with a constant step of 0.02°at room temperature (see Supplementary Fig. 1). Temperature-variable XRD patterns were collected on a Rigaku Smartlab diffractometer with Cu-*K*_α_ radiation. A polycrystalline sample was cooled down to 175 K, where it was kept for 30 min. Then, the measurements were performed over 175–375 K for every 10 K. All patterns were analyzed with Rietveld refinement method using the FullProf suites^61^.

### Barocaloric measurements

The heat flow measurements were performed using a high-pressure differential scanning calorimeter, µDSC, Setaram^8,62,63^. For the constant-pressure measurements as a function of temperature, a powder sample weighted ∼20 mg was sealed into a high-pressure vessel made of Hastelloy, while an empty vessel was also used as a reference. Desirable hydrostatic pressures were generated and maintained by controlling argon gas pressure through the high-pressure gas panel. The data were collected in the temperature range from 230 to 340 K under the constant pressure of 0.1, 10, 20, 30, 40, 50, 60, 70, and 80 MPa, respectively. A ramping rate of 1 K min^−1^ was used for both the cooling and heating processes. For variable-pressure measurements at room temperature, a powder sample weighted ∼42 mg was sealed into the Hastelloy vessel. The heat flow variations with time were recorded for the pressurization (50–90 MPa) and depressurization (50–7.5 MPa) processes, as drawn in Supplementary Fig. 5. The background was determined by avoiding phase transitions, which was guaranteed by the thermal hysteresis.

### INS/QENS measurements

The INS and/or QENS experiments were conducted using the cold-neutron time-of-flight spectrometer, PELICAN, at the Australian Centre for Neutron Scattering, ANSTO^44^. The instrument was configured with an incident neutron wavelength of 4.69 Å, affording incident energy of 3.72 meV with an energy resolution of 0.135 meV at the elastic line. A powder sample was sealed into an annular aluminum can. The measurements were carried out from 100 to 390 K to cover the two-phase transitions. The empty can was measured in the same conditions for background subtraction. In addition, a standard vanadium sample was also measured for detector normalization and determination of the energy resolution function. The high-pressure neutron scattering measurements were performed with the same configuration at 0.1 and 300 MPa at 300 K. A bespoke high-pressure cell made of Be-Cu alloys was used, and a KBr powder sample was employed to calibrate the actual pressures. The data reduction, including background subtraction and detector normalization, was performed using the Large Array Manipulation Program (LAMP)^64^, while the sliced QENS spectra were analyzed in the Pan module built-in the Data Analysis and Visualization Environment (DAVE)^65^.

### Analysis of the reorientation dynamics

In the INS spectrum, the broadening underneath the elastic peak is the signal of QENS, which is associated with diffusive and/or reorientation motions. Assuming one single reorientation mode, the QENS dynamics structural factor $S\left(Q,\omega \right)$ can be described as^66^:

1 $$S\left(Q,\omega \right)=f\times \left[{A}_{{{{{{\rm{E}}}}}}}\left(Q\right)\delta \left(\omega \right)+\sum {A}_{{{{{{\rm{QE}}}}}},i}\left(Q\right){L}_{i}\left(Q,\omega \right)\right]\otimes R\left(Q,\omega \right)+b\left(Q,\omega \right)$$

where $\delta \left(\omega \right)$ is a delta function representing the elastic peak, ${A}_{{{{{{\rm{E}}}}}}}\left(Q\right)$ and ${A}_{{{{{{\rm{QE}}}}}}}\left(Q\right)$ are the weights of the elastic and quasi-elastic scattering, *f* is a scaling factor, $R\left(Q,\omega \right)$ is experimentally determined resolution function, and $b\left(Q,\omega \right)$ is a linear background. The symbol ⨂ describes numerical convolution between the elastic/quasi-elastic components and instrumental resolution. The quasielastically broadened energy distribution could be well depicted by a Lorentzian function:

2 $$L\left(Q,\omega \right)=\frac{1}{\pi }\frac{\frac{1}{2}\varGamma \left(Q\right)}{{\left({{\hslash }}\omega \right)}^{2}+{\left(\frac{1}{2}\varGamma \left(Q\right)\right)}^{2}}$$

where *Γ*(*Q*) is the full width at half maximum, corresponding to the frequency of a motion.

To analyze the reorientation geometry of the [NH_4_]^+^ tetrahedra, EISF was extracted from spectral fitting:

3 $${{{{{\rm{EISF}}}}}}=\frac{{A}_{{{{{{\rm{E}}}}}}}\left(Q\right)}{{A}_{{{{{{\rm{E}}}}}}}\left(Q\right)+{A}_{{{{{{\rm{QE}}}}}}}\left(Q\right)}$$

Three models were considered to reproduce the experimentally determined EISF^67^:

*C*_2_ and/or *C*_3_ jumps:

4 $${{{{{\rm{EISF}}}}}}=\frac{1}{2}\left[1+{j}_{0}\left(Qd\right)\right]$$

Cubic tumbling:

5 $${{{{{\rm{EISF}}}}}}=\frac{1}{8}\left[1+3{j}_{0}\left({Qd}/\sqrt{2}\right)+3{j}_{0}\left({Qd}\right)+{j}_{0}\left({Qd}\sqrt{3/2}\right)\right]$$

Isotropic rotational diffusion:

6 $${{{{{\rm{EISF}}}}}}={j}_{0}^{2}\left({Qr}\right)$$

where *d* is the jump distance, *r* is the rotational radius, and ${j}_{0}\left(x\right)=\frac{{\sin }\left(x\right)}{x}$ is the spherical Bessel function of the zeroth order^67^. In the case of NH_4_I, *d* is close to the H–H distance in the [NH_4_]^+^ tetrahedra (∼1.67 Å), whereas *r* is the same as the N–H bond length or $r=d\times \sqrt{3/8}$ (∼1.02 Å)^68^.

When the possible orientational geometries at a phase transition are determined, the configurational entropies across this phase transition can be estimated by the formula as follows:

7 $$\triangle {S}_{{{{{{\rm{conf}}}}}}}=R{{{{{\rm{ln}}}}}}\frac{{N}_{2}}{{N}_{1}}$$

where *R* is the gas constant, *N*_1_ and *N*_2_ are the numbers of possible orientational configurations below and above a phase transition temperature.

### Analysis of the lattice dynamics

The measured dynamic structure factor with powder sample correlates to the phonon DOS, *g*(*E*)^69^:

8 $$g\left(E\right)=A\times \left\langle 4M\frac{{{\exp }}\left(2W\right)}{{{{\hslash }}}^{2}{Q}^{2}}\frac{E}{n\left(E,T\right)+\frac{1}{2}\pm \frac{1}{2}}S\left(Q,\omega \right)\right\rangle$$

where *A* is a scaling factor, *M*, and ${{\exp }}\left(-2W\right)$ are the atomic mass and Debye-Waller factor, respectively, while the ‘+’ and ‘–‘ signs denote energy loss or energy gain of neutrons, respectively. $n\left(E,T\right)$ is the Bose-Einstein occupation factor, defined as $n\left(E,T\right)={\left[{{\exp }}\left(E/{k}_{B}T\right)-1\right]}^{-1}$, where *k*_B_ is the Boltzmann constant. The brackets 〈⋯〉 represent the average operator over all *Q* ranges at a given energy. In a polyatomic material, the experimental determined phonon DOSs are neutron-weighted as^70^,

9 $${g}_{{{{{{\rm{NW}}}}}}}\left(E\right)=\mathop{\sum}\limits_{i}{f}_{i}\frac{{\sigma }_{i}}{{M}_{i}}{g}_{i}\left(E\right){{\exp }}\left(-2{W}_{i}\right)$$

here *i* represents different elements, *f*_*i*_ is the atomic concentration, ${g}_{i}\left(E\right)$ is the real partial phonon DOS of the element *i*. In this work, due to the limited maximum value (∼3.72 meV) on the energy loss side, only the energy gain side of the $S\left(Q,\omega \right)$ up to 80 meV was used to extract the ${g}_{{{{{{\rm{NW}}}}}}}\left(E\right)$. In addition, it is noted that the values of $\sigma /M$ for N, H, and I are 0.82, 82.02, and 0.03 barn/amu, respectively. Therefore, the obtained ${g}_{{{{{{\rm{NW}}}}}}}\left(E\right)$ mainly reflects the vibrational information of hydrogen atoms.

### Reporting summary

Further information on research design is available in the Nature Research Reporting Summary linked to this article.

## Supplementary information

Supplementary information

Reporting Summary

## Data Availability

The data that support the findings of this study are available from the corresponding author upon request.
