# Supplementary material for: Ultrasensitive barocaloric material for room-temperature solid-state refrigeration
Source: Nat Commun. 2022 Apr 28;13:2293. doi: 10.1038/s41467-022-29997-9 (PMC9051211; doi:10.1038/s41467-022-29997-9)
Supplement: Supplementary file 1 — Supplementary information [file 41467_2022_29997_MOESM1_ESM.pdf]

Supplementary Materials

Ultrasensitive barocaloric material for room-temperature solid-state refrigeration

Qingyong Ren[1,2,#], Ji Qi[3,4,#], Dehong Yu[5], Zhe Zhang[3,4], Ruiqi Song[3], Wenli Song[1,2], Bao Yuan[1,2], Tianhao Wang[1,2], Weijun Ren[3], Zhidong Zhang[3,4], Xin Tong[1,2,]* & Bing Li[3,4,]*

[1]Institute of High Energy Physics, Chinese Academy of Sciences, Beijing 100049, China
[2]Spallation Neutron Source Science Center, Dongguan 523803, China
[3]Shenyang National Laboratory for Materials Science, Institute of Metal Research, Chinese Academy of Sciences, 72 Wenhua Road, Shenyang 110016, China.
[4]School of Materials Science and Engineering, University of Science and Technology of China, 72 Wenhua Road, Shenyang 110016, China.
[5]Australian Nuclear Science and Technology Organisation, Lucas Heights, New South Wales 2234, Australia.

Correspondence and requests for materials should be addressed to X.T. (email: tongx@ihep.ac.cn) or to B.L. (email: bingli@imr.ac.cn).

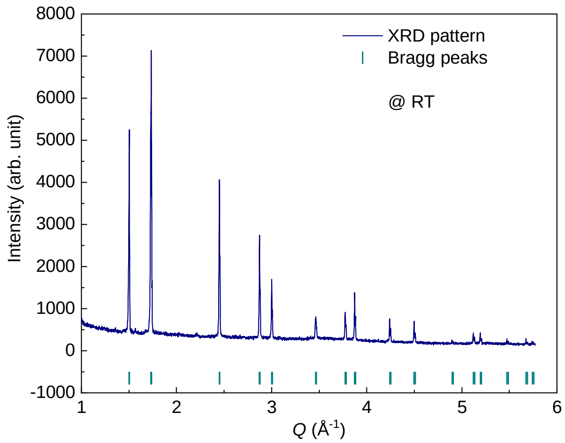

Supplementary Fig. 1. X-ray diffraction (XRD) pattern of an NH_4I powder sample at room temperature (RT). The sample crystallized in the high-T α-phase with a space group of $Fm\bar{3}m$.

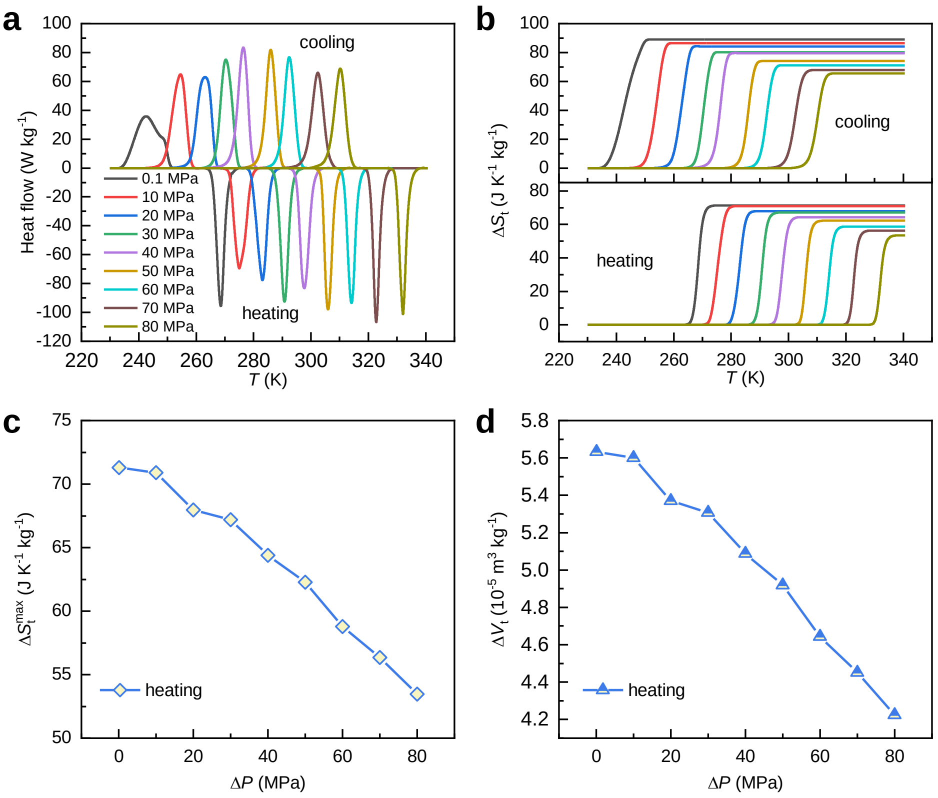

Supplementary Fig. 2 Calorimetric data of NH_4I. a, Heat flow on cooling and heating with different pressure in the temperature range from 230 to 340 K. Even if the cooling curve under ambient pressure exhibits a 'two-step broad peak' feature, the integration of this peak is reasonable in comparison with the integration values with higher pressure. Its occurrence might be associated with the thermal history of the NH_4I compound. **b**, Entropy changes of the phase transition as a function of temperature under constant pressures. **c**,**d**, The maximum isobaric entropy changes $\Delta S_{\mathrm{t}}^{\mathrm{max}}$ and volume change ΔV_{t} at the $\beta \rightarrow \alpha$ phase transition. It is noted that $\Delta S_{\mathrm{t}}^{\mathrm{max}}$ decreases with increasing pressure. This decreasing trend of $\Delta S_{\mathrm{t}}^{\mathrm{max}}(P)$ implies a reduced volume change of phase transition, $\Delta V_{\mathrm{t}}(P)$, with the application of external pressure. Far away from the phase transition, the entropy change due to the lattice contraction is estimated to be smaller than 0.4 J kg^{-1} K^{-1} in terms of $-(\partial V/\partial T)_{P=0} \times P$ (see Supplementary Fig. S7 for more details)[1, 2].

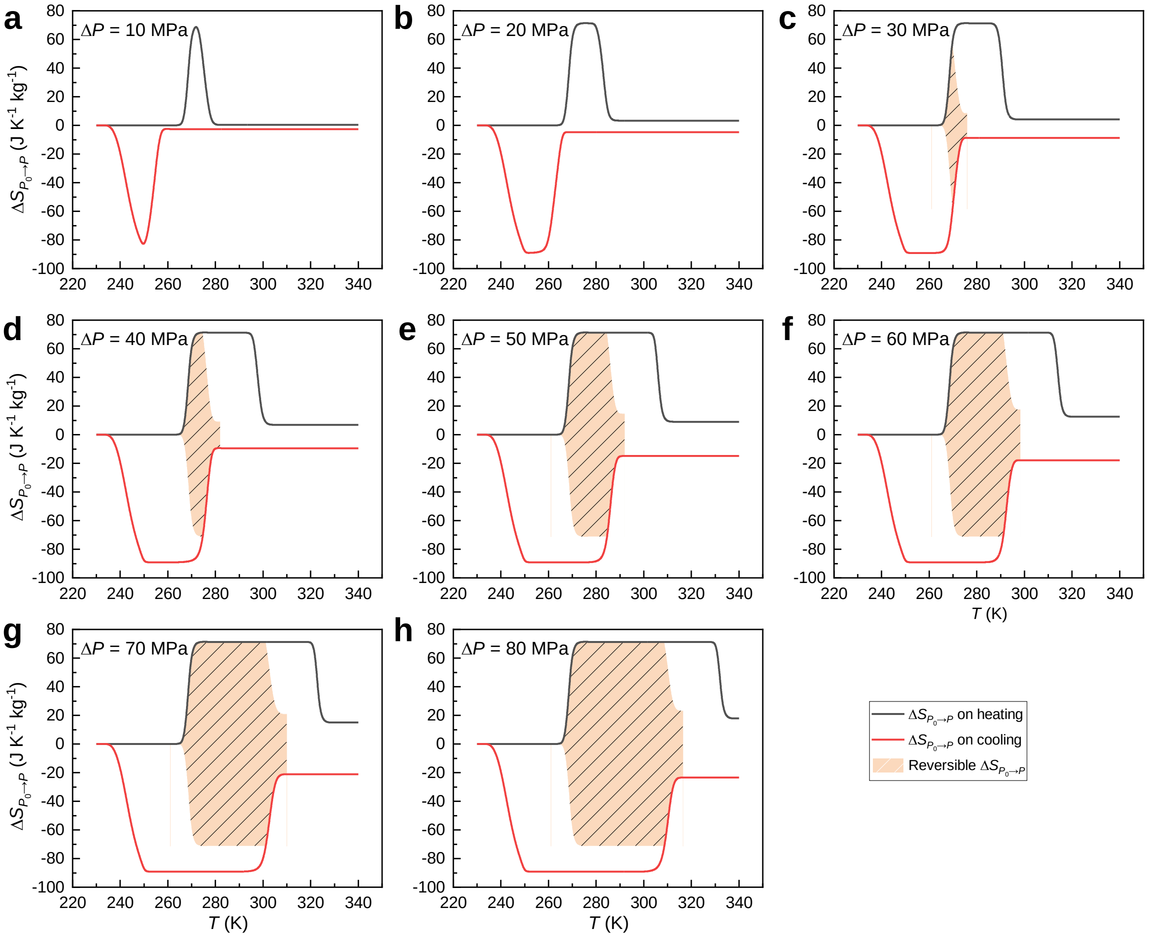

Supplementary Fig. 3 Reversibility of the barocaloric effect of NH_4I. a-h, The pressure-induced entropy change, $\Delta S_{P_0 \to P}$, with the reversible part marked by the shaded areas.

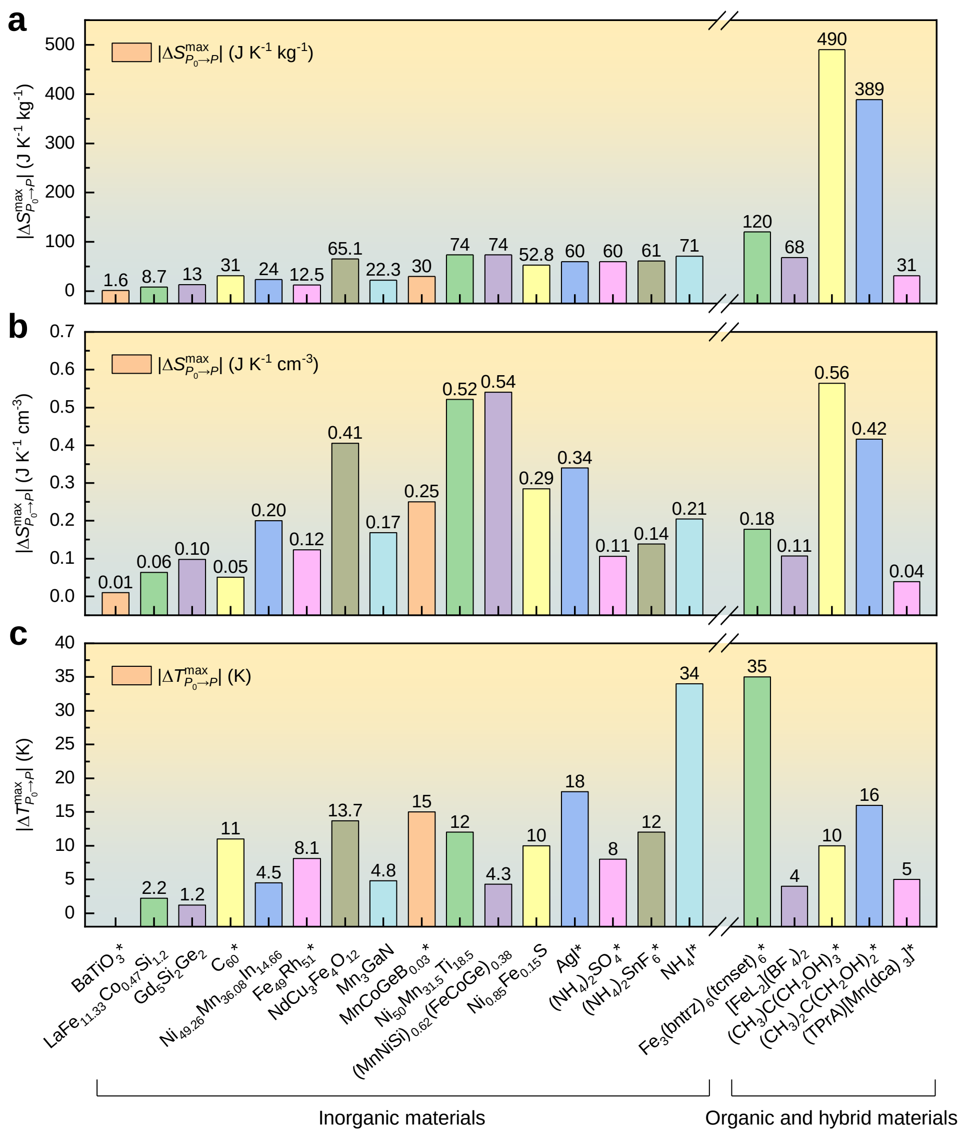

Supplementary Fig. 4 Comparison of the barocaloric performances of NH_4I. a, Maximum entropy changes in J kg^{-1} K^{-1}. **b**, Maximum entropy changes in J cm^{-3} K^{-1}. **c**, Maximum adiabatic temperature changes calculated from the entropy changes with the formula of $\left|T\Delta S_{P_0\to P}/C_P\right|$. The data for other samples are cited elsewhere[1-21], and reversible values are marked with '*'.

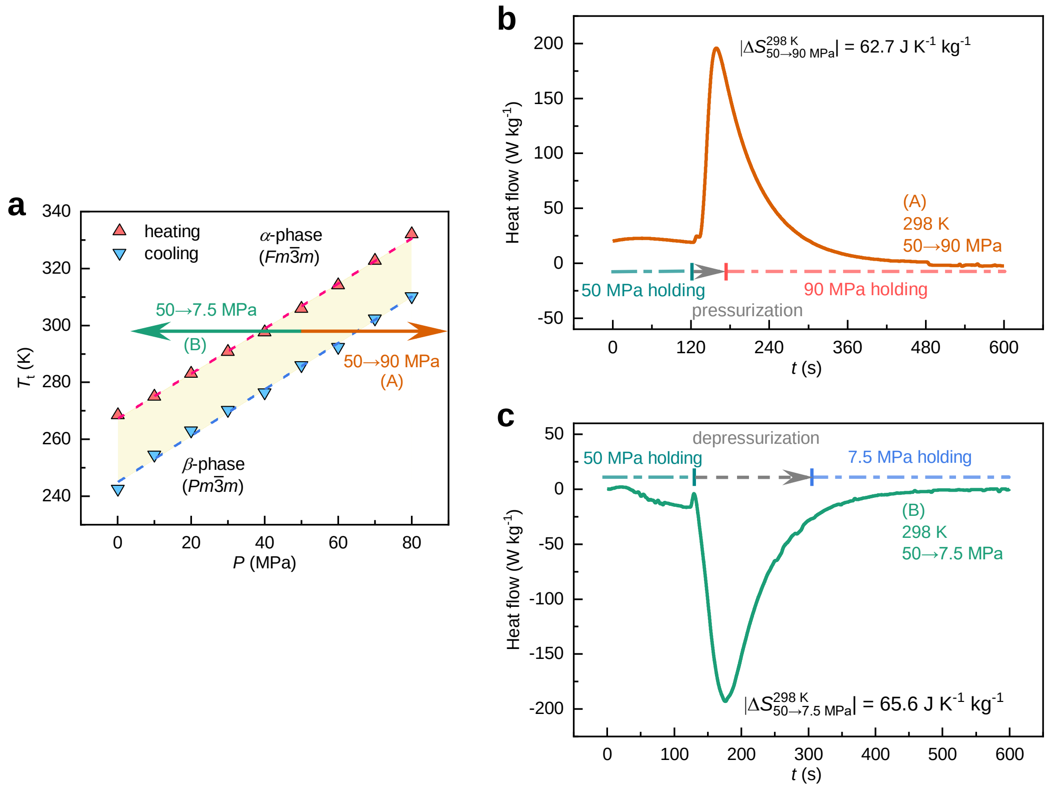

Supplementary Fig. 5 In situ pressure-induced heat flow. a, The schematic figure for pressurization and depressurization processes as arrowed in the phase diagram of NH_4I. **b**,**c**, Heat flow of NH_4I as a function of time for the pressurization (50 → 90 MPa) and depressurization (50 → 7.5 MPa) processes, respectively. The detailed pressurization and depressurization processes are marked by the dashed lines. The pressure-driven entropy changes are estimated by integrating the heat flow curves.

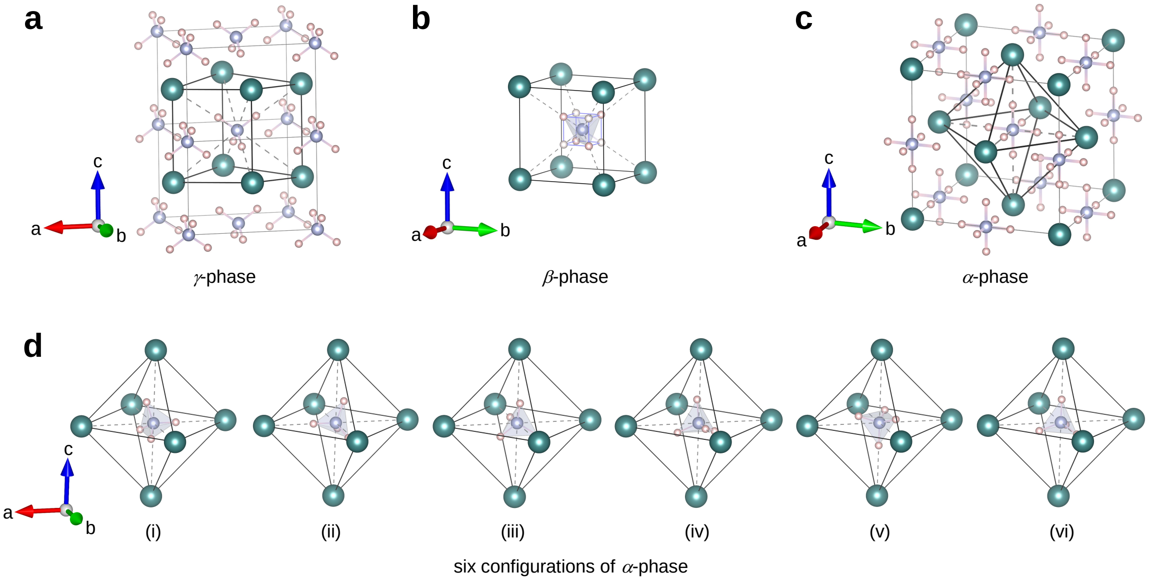

Supplementary Fig. 6 The crystal structures of NH_4I. a, low-*T* γ-phase with the space group of *P4/nmm*, **b**, Intermediate-*T* β-phase with $Pm\bar{3}m$ structure, and **c**,**d** high-*T* α-phase with $Fm\bar{3}m$ structure[22-24]. In the crystal structure of the α-phase, the single-approach model is used, in which one of the N-H bonds of the $[NH_4]^+$ tetrahedra is aligned along the [100] direction so that only one H atom in the $[NH_4]^+$ tetrahedra can approach to the I^- anions as shown in (**d**)[24]. Following the symmetric operation of the α-phase, there exist six reorientations in this configuration (without considering any possible rotation about this N-H bond). The six hydrogen atoms in (**c**) around each N atom represent the six possible positions of the single-approach H atom of the tetrahedron, and the steric orientations of $[NH_4]^+$ tetrahedra for each single-approach hydrogen atom in (**c**) are shown in (**d**).

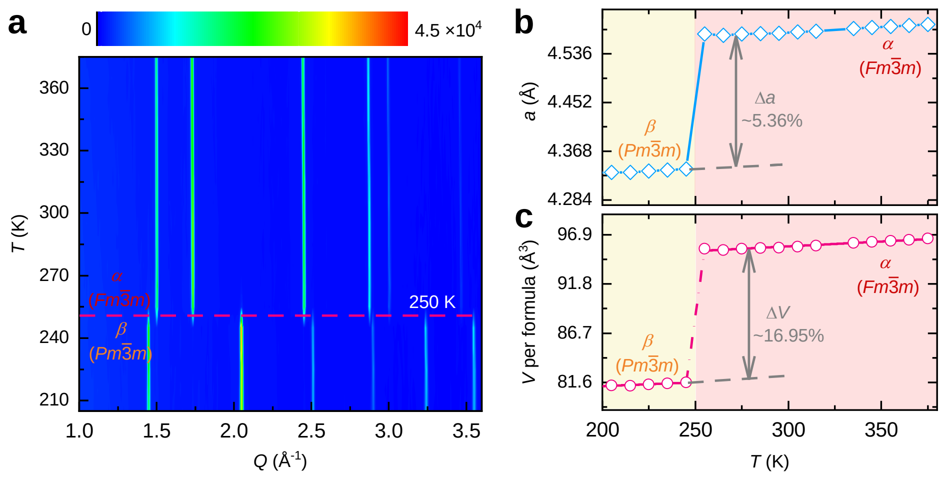

Supplementary Fig. 7 Temperature dependent XRD patterns and lattice parameters. a, The contour plot of the XRD patterns in the temperature region from 205 to 375 K. **b**,**c**, The variations of the lattice parameter (a) and the unit cell volume per formula (V) with temperature. The temperature dependence of the volume, $(\partial V/\partial T)_{P=0}$, for the intermediate-$T$ β-phase is 3.30×10^{-8} m^3 kg^{-1} K^{-1}, whereas 4.23×10^{-8} m^3 kg^{-1} K^{-1} for the high-T α-phase. Then, the entropy changes due to the lattice contraction can be estimated via the formula of $-(\partial V/\partial T)_{P=0} \times P$ (here, the specific heat and $(\partial V/\partial T)_{P=0}$ are assumed to be pressure independent)[1, 2]. The entropy changes due to lattice contraction at 80 MPa for the β and α phases are determined as 0.26 J kg^{-1} K^{-1} and 0.34 J kg^{-1} K^{-1}, respectively. These two values are much smaller than the entropy changes $\left|\Delta S_{P_0 \to P}\right|$ as depicted in Fig. 1b.

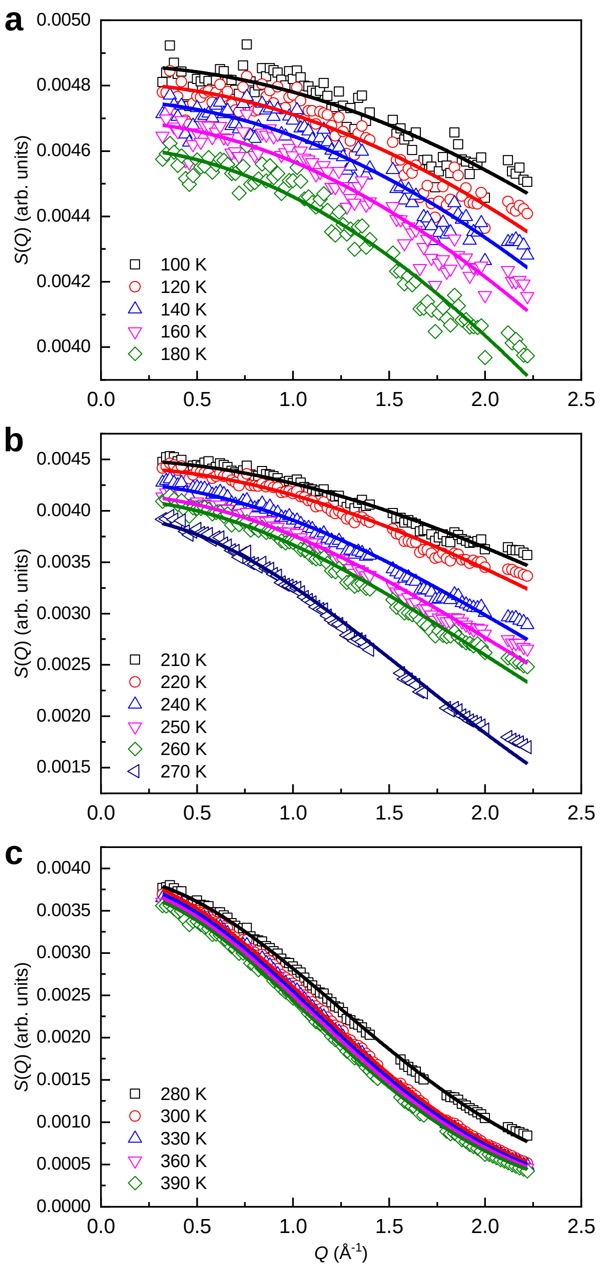

Supplementary Fig. 8 The fitting of the elastic structure factor, $S(Q)$, following the Debye-Waller effect, $S(Q) \propto \exp(-Q^2\langle u^2\rangle/3)$ [25], to determine the mean squared displacements over the temperature range from 100 to 390 K. The Bragg peaks are excluded in the fitting.

Supplementary Table 1. A comparison of barocaloric performances of leading materials.

Sample	$\lvert dT_t/dP \rvert$ [K MPa^{-1}]	ΔP [MPa]	$\lvert \Delta S^{max}_{P_0 \to P} \rvert$ [J K^{-1} kg^{-1}]	$\lvert \Delta S^{max}_{P_0 \to P}/\Delta P \rvert$ [J K^{-1} kg^{-1} MPa^{-1}]	Density [g cm^{-3}]	$\lvert \Delta S^{max}_{P_0 \to P} \rvert$ [J K^{-1} cm^{-3}]	$\lvert \Delta T_{ad} \rvert$ [K]	T_t [K]	Ref.
$BaTiO_3$*	0.06	100	1.6$^{*, qd}$	0.02	6.024	0.010	-	400	[3]
$LaFe_{11.33}Co_{0.47}Si_{1.2}$	0.01	200	8.7^{qd}	0.04	7.2	0.063	2.2^{dir}	237	[4]
$Gd_5Si_2Ge_2$	0.04	290	13^{qd}	0.04	7.54	0.098	1.2^{dir}	270	[5]
C_{60}*	0.17	410	31$^{*, qd}$	0.08	1.65	0.051	11$^{*, ind}$	255	[14]
$Ni_{49.26}Mn_{36.08}In_{14.66}$	0.02	260	24^{qd}	0.09	8.2	0.200	4.5^{ind}	293	[8]
$Fe_{49}Rh_{51}$*	0.06	110	12.5$^{*, qd}$	0.11	9.8	0.123	8.1$^{*, ind}$	308	[6, 21]
$NdCu_3Fe_4O_{12}$	0.04	510	65.1^{qd}	0.13	6.225	0.405	13.7^{ind}	294	[20]
Mn_3GaN	0.07	139	22.3^{ind}	0.16	7.6	0.169	4.8^{ind}	285	[7]
$MnCoGeB_{0.03}$*	0.1	170	30$^{*, qd}$	0.18	7.65	0.25	~15^{ind}	286	[9]
$Ni_{50}Mn_{31.5}Ti_{18.5}$	0.03	400	74^{qd}	0.19	7.04	0.521	~12^{ind}	243	[12]
$(MnNiSi)_{0.62}(FeCoGe)_{0.38}$	0.08	270	74^{qd}	0.27	7.3	0.540	4.3^{dir}	338	[11]
$Ni_{0.85}Fe_{0.15}S$	0.08	100	52.8^{qd}	0.53	5.69	0.285	10^{ind}	303	[10]
AgI*	0.14	250	60$^{*, qd}$	0.24		0.34	18$^{*, ind}$	420	[1]
$(NH_4)_2SO_4$*	0.05	100	60$^{*, qd}$	0.60	1.7	0.106	8$^{*, ind}$	219	[2]
$(NH_4)_2SnF_6$*	0.16	100	61$^{*, qd}$	0.61	2.271	0.139	~12^{ind}	105	[16]
NH_4I*	0.81	40	71$^{*, qd}$	1.78	2.89	0.205	34^{ind}	275	This work
$Fe_3(bntrz)_6(tcnset)_6$*	0.25	260	120$^{*, qd}$	0.46	1.485	0.178	35$^{*, ind}$	318	[17]
$[FeL_2](BF_4)_2$	0.15	43	68^{qd}	1.58		0.107	4^{ind}	258	[15]
$(CH_3)C(CH_2OH)_3$*	0.08	240	490$^{*, qd}$	2.04	1.15	0.564	10$^{*, ind}$	354	[18, 19]
$(CH_3)_2C(CH_2OH)_2$*	0.12	90	389$^{*, qd}$	4.32	1.069	0.416	16$^{*, ind}$	315	[18, 19]
$(TPrA)[Mn(dca)_3]$*	0.23	7	31$^{*, qd}$	4.43	1.265	0.039	5$^{*, ind}$	330	[13]

$\lvert dT_t/dP \rvert$, pressure dependence of phase transition temperature, T_t;

ΔP, saturated driving pressure to meet the maximum isothermal entropy change, $\lvert \Delta S^{max}_{P_0 \to P} \rvert$;

$\lvert \Delta S^{max}_{P_0 \to P}/\Delta P \rvert$, barocaloric strength, defined by maximum isothermal entropy change normalized by saturation pressure;

$\lvert \Delta T_{ad} \rvert$ is the adiabatic temperature change.

All data marked with "*" represent the reversible value. $\lvert \Delta S^{max}_{P_0 \to P} \rvert$ value for most samples are obtained using a quasi-direct (qd) method, except Mn_3GaN where a Maxwell (indirect, "ind") method is used. "dir" denotes $\lvert \Delta T_{ad} \rvert$ data derived from direct measurement, while other $\lvert \Delta T_{ad} \rvert$ data obtained through $\Delta T_{ad} = \lvert T \Delta S_{P_0 \to P}/C_P \rvert$ (C_P is the constant-pressure heat capacity) are also denoted as indirect method ("ind").

References:

1. Aznar, A., *et al.* Giant barocaloric effects over a wide temperature range in superionic conductor AgI. *Nat. Commun.* **8**, 1851 (2017).

2. Lloveras, P., *et al.* Giant barocaloric effects at low pressure in ferrielectric ammonium sulphate. *Nat. Commun.* **6**, 8801 (2015).

3. Stern-Taulats, E., *et al.* Inverse barocaloric effects in ferroelectric $BaTiO_3$ ceramics. *APL Mater.* **4**, 091102 (2016).

4. Manosa, L., *et al.* Inverse barocaloric effect in the giant magnetocaloric La-Fe-Si-Co compound. *Nat. Commun.* **2**, 595 (2011).

5. Yuce, S., *et al.* Barocaloric effect in the magnetocaloric prototype $Gd_5Si_2Ge_2$. *Appl. Phys. Lett.* **101**, 071906 (2012).

6. Stern-Taulats, E., *et al.* Barocaloric and magnetocaloric effects in $Fe_{49}Rh_{51}$. *Phys. Rev. B* **89**, 214105 (2014).

7. Matsunami, D., *et al.* Giant barocaloric effect enhanced by the frustration of the antiferromagnetic phase in Mn_3GaN. *Nat. Mater.* **14**, 73-78 (2015).

8. Manosa, L., *et al.* Giant solid-state barocaloric effect in the Ni-Mn-In magnetic shape-memory alloy. *Nat. Mater.* **9**, 478-481 (2010).

9. Aznar, A., *et al.* Giant and Reversible Inverse Barocaloric Effects near Room Temperature in Ferromagnetic $MnCoGeB_{0.03}$. *Adv. Mater.* **31**, e1903577 (2019).

10. Lin, J., *et al.* Giant room-temperature barocaloric effect at the electronic phase transition in $Ni_{1-x}Fe_xS$. *Mater. Horizons* **7**, 2690-2695 (2020).

11. Samanta, T., *et al.* Barocaloric and magnetocaloric effects in $(MnNiSi)_{1-x}(FeCoGe)_x$. *Appl. Phys. Lett.* **112**, 021907 (2018).

12. Aznar, A., *et al.* Giant barocaloric effect in all-d-metal Heusler shape memory alloys. *Phys. Rev. Mater.* **3**, 044406 (2019).

13. Bermudez-Garcia, J. M., *et al.* Giant barocaloric effect in the ferroic organic-inorganic hybrid [TPrA][Mn(dca)$_3$] perovskite under easily accessible pressures. *Nat. Commun.* **8**, 15715 (2017).

14. Li, J., *et al.* Reversible barocaloric effects over a large temperature span in fullerite C_{60}. *J. Mater. Chem. A* **8**, 20354-20362 (2020).

15. Vallone, S. P., *et al.* Giant Barocaloric Effect at the Spin Crossover Transition of a Molecular Crystal. *Adv. Mater.* **31**, e1807334 (2019).

16. Flerov, I. N., *et al.* Thermal, structural, optical, dielectric and barocaloric properties at ferroelastic phase transition in trigonal $(NH_4)_2SnF_6$: A new look at the old compound. *J. Fluorine Chem.* **183**, 1-9 (2016).

17. Romanini, M., *et al.* Giant and Reversible Barocaloric Effect in Trinuclear Spin-Crossover Complex $Fe_3(bntrz)_6(tcnset)_6$. *Adv. Mater.* **33**, 2008076 (2021).

18. Aznar, A., *et al.* Reversible and irreversible colossal barocaloric effects in plastic crystals. *J. Mater. Chem. A* **8**, 639-647 (2020).

19. Li, B., *et al.* Colossal barocaloric effects in plastic crystals. *Nature* **567**, 506-510 (2019).

20. Kosugi, Y., *et al.* Colossal Barocaloric Effect by Large Latent Heat Produced by First-Order Intersite-Charge-Transfer Transition. *Adv. Funct. Mater.* **31**, 2009476 (2021).

21. Stern-Taulats, E., *et al.* Reversible adiabatic temperature changes at the magnetocaloric and barocaloric effects in $Fe_{49}Rh_{51}$. *Appl. Phys. Lett.* **107**, 152409 (2015).

22. Levy, H. A. & Peterson, S. W. Neutron Diffraction Determination of the Crystal Structure of Ammonium Bromide in Four Phases1. *J. Am. Chem. Soc.* **75**, 1536-1542 (1953).

23. Durig, J. R. & Antion, D. J. Low-Frequency Vibrations in Ammonium Iodide and Ammonium Bromide. *J. Chem. Phys.* **51**, 3639-3647 (1969).

24. Levy, H. A. & Peterson, S. W. Neutron Diffraction Study of the NaCl-type Modification of ND_4Br and ND_4I. *J. Chem. Phys.* **21**, 366-366 (1953).

25. Telling, M. T. F., *et al.* Anharmonic Behavior in the Multisubunit Protein Apoferritin as Revealed by Quasi-Elastic Neutron Scattering. *J. Phys. Chem. B* **112**, 10873-10878 (2008).
