# Supplementary material for: Ultrasensitive barocaloric material for room-temperature solid-state refrigeration
Source: Nat Commun. 2022 Apr 28;13:2293. doi: 10.1038/s41467-022-29997-9 (PMC9051211; doi:10.1038/s41467-022-29997-9)
Supplement: Supplementary file 2 — Reporting Summary [file 41467_2022_29997_MOESM2_ESM.pdf]

nature portfolio

Corresponding author(s): Bing Li, Xin Tong

Last updated by author(s): Mar 30, 2022

Reporting Summary

Nature Portfolio wishes to improve the reproducibility of the work that we publish. This form provides structure for consistency and transparency in reporting. For further information on Nature Portfolio policies, see our Editorial Policies and the Editorial Policy Checklist.

Statistics

For all statistical analyses, confirm that the following items are present in the figure legend, table legend, main text, or Methods section.

n/a	Confirmed	
☒	☐	The exact sample size (*n*) for each experimental group/condition, given as a discrete number and unit of measurement
☒	☐	A statement on whether measurements were taken from distinct samples or whether the same sample was measured repeatedly
☒	☐	The statistical test(s) used AND whether they are one- or two-sided<br>*Only common tests should be described solely by name; describe more complex techniques in the Methods section.*
☒	☐	A description of all covariates tested
☒	☐	A description of any assumptions or corrections, such as tests of normality and adjustment for multiple comparisons
☒	☐	A full description of the statistical parameters including central tendency (e.g. means) or other basic estimates (e.g. regression coefficient) AND variation (e.g. standard deviation) or associated estimates of uncertainty (e.g. confidence intervals)
☒	☐	For null hypothesis testing, the test statistic (e.g. *F*, *t*, *r*) with confidence intervals, effect sizes, degrees of freedom and *P* value noted<br>*Give P values as exact values whenever suitable.*
☒	☐	For Bayesian analysis, information on the choice of priors and Markov chain Monte Carlo settings
☒	☐	For hierarchical and complex designs, identification of the appropriate level for tests and full reporting of outcomes
☒	☐	Estimates of effect sizes (e.g. Cohen's *d*, Pearson's *r*), indicating how they were calculated

Our web collection on statistics for biologists contains articles on many of the points above.

Software and code

Policy information about availability of computer code

Data collection	no software was used
Data analysis	DAVE, LAMP, PAN

For manuscripts utilizing custom algorithms or software that are central to the research but not yet described in published literature, software must be made available to editors and reviewers. We strongly encourage code deposition in a community repository (e.g. GitHub). See the Nature Portfolio guidelines for submitting code & software for further information.

Data

Policy information about availability of data

All manuscripts must include a data availability statement. This statement should provide the following information, where applicable:

- Accession codes, unique identifiers, or web links for publicly available datasets
- A description of any restrictions on data availability
- For clinical datasets or third party data, please ensure that the statement adheres to our policy

The data that support the findings of this study are available from the corresponding author upon request.

Field-specific reporting

Please select the one below that is the best fit for your research. If you are not sure, read the appropriate sections before making your selection.

☐ Life sciences ☐ Behavioural & social sciences ☒ Ecological, evolutionary & environmental sciences

For a reference copy of the document with all sections, see nature.com/documents/nr-reporting-summary-flat.pdf

Ecological, evolutionary & environmental sciences study design

All studies must disclose on these points even when the disclosure is negative.

Study description	In this work, we mainly studied the barocaloric effects of NH4I as well as its crystal structures and dynamics. All the experimental measurements in this study have been done based on standard instruments or devices, and the experimental results are highly reproducible.
Research sample	The sample studied in this manuscript was carefully selected by the comment requirement for large barocaloric effect materials, including its crystal structures, proper phase transition temperature around room temperature as well as ability to be driven by pressure. Through literature search, we found that NH4I compound might be a good candidate, and finally taken it as the sample in this study.
Sampling strategy	In this work, only one NH4I compound were selected. For caloric, neutron scattering and x-ray diffraction measurements, the sample masses are carefully selected based on the requirement of the instruments or techniques.
Data collection	For caloric measurements were carried out mainly by Ji Qi.<br>The X-ray diffraction measurements were collected by the Hangzhou Yanqu Information Technology Co., Ltd.<br>The neutron scattering measurements were carried out at ANSTO by Dehong Yu, Qingyong Ren, Wenli Song and Bing Li.<br>All details about these measurements have been provided in the manuscript.
Timing and spatial scale	For caloric measurements were carried out over Feb. 2020 to March 2022. This including the data in the first version and revised versions as responses to reviewers' comments.<br>The X-ray diffraction measurements were collected over November 27-29.<br>The neutron scattering measurements were carried out at October 2020.
Data exclusions	No data was exclued from the analysis.
Reproducibility	The barocaloric effect reported in this work were checked with different methods, and all these results are in good agreement. This confirm the reproducibility of the experimental finding in this work.
Randomization	In this work, only one compound was studied. It does not need to take any randomization.
Blinding	Not applicable.

Did the study involve field work? ☐ Yes ☒ No

Reporting for specific materials, systems and methods

We require information from authors about some types of materials, experimental systems and methods used in many studies. Here, indicate whether each material, system or method listed is relevant to your study. If you are not sure if a list item applies to your research, read the appropriate section before selecting a response.

Materials & experimental systems

n/a	Involved in the study	
☒	☐	Antibodies
☒	☐	Eukaryotic cell lines
☒	☐	Palaeontology and archaeology
☒	☐	Animals and other organisms
☒	☐	Human research participants
☒	☐	Clinical data
☒	☐	Dual use research of concern

Methods

n/a	Involved in the study	
☒	☐	ChIP-seq
☒	☐	Flow cytometry
☒	☐	MRI-based neuroimaging
